# Supplementary material for: Agent-based modeling demonstrates how target-independent processes supplement killing by antibody-drug conjugates in cancer therapy
Source: PLoS Comput Biol. 2026 May 26;22(5):e1013872. doi: 10.1371/journal.pcbi.1013872 (PMC13274927; doi:10.1371/journal.pcbi.1013872)
Supplement: S2 Text — (DOCX) [file pcbi.1013872.s002.docx]

Commonly used parameters in the *SimADC* range.xml file. Current values represent simulating T-DXd in a low expression NCI-N87 tumor with macrophage uptake and payload release.

**initTumorVolume** **–** Initial tumor volume (mm^3^)

Cancer cell growth parameters (up to 4 different types of cancer cells can be simulated with varying drug sensitivity, growth rate, and expression level):

**initPayloadTargetFreeNanoMoles** – intracellular payload target concentration (nM)

**maxKillProb** – maximum killing probability

**michaelisConstant** – Michaelis-Menten constant, Km (nM)

**minKillingConc** – minimum concentration necessary for cell death (nM)

**doublingTimeParamA, doublingTimeParamB** – slope and y-intercept of linear relationship between blood vessel density and doubling time

**doublingTimeMin + doublingTimeMax** – min and max cancer cell doubling times (timesteps)

**receptorsPerCellMean** – cancer cell receptor expression (number of receptors)

Cd8 T cell parameters:

**activeInitDensity, inactiveInitDensity** – psercentage of active/inactive T cells on the grid

**activeProbKill, inactiveProbKill** – killing probabilities of active/inactive T cells

Macrophage parameters:

**initDensity** – percentage of macrophages on the grid

**receptorsPerCellMean** – Fc receptor expression on macrophages (number of receptors)

**activeFraction** – active fraction of blood vessels

Antibody parameters (for cancer cells and macrophages):

**kOn** – antibody binding on rate (1/nM-s)

**Kd** – binding affinity (nM)

**ke –** receptor internalization rate (1/s)

**keBound** – bound complex internalization rate (1/s)

Payload-specific parameters (for cancer cells and macrophages):

**kInP** – payload permeability rate into cell (1/s)

**kOutP** – payload permeability rate out of cell (1/s)

**kOnP** – payload binding rate to intracellular target (1/M-s)

**kOffP** – payload unbinding rate from intracellular target (1/s)

Pharmacokinetic parameters:

**clearanceTotalAntibody** – total antibody clearance (1/s)

**clearanceCompartment12** – clearance from compartment 1 to 2 (1/s)

**clearanceCompartment21** – clearance from compartment 2 to 1 (1/s)

Dosing parameters: (Drug1 = ADC, Drug2 = free payload, Drug3 = unconjugated antibody)

**doseConcentration** – drug dose (mg/kg)

Extracellular cleavage parameters:

**kCleaveFree, kCleaveBound** – rate of extracellular cleavage for free and bound ADC (1/s)

Commonly used options and values for the *SimADC* executable. Current values are typical of a simulated *in vivo* tumor treated with ADC.

**--dim 600** – grid dimensions

**--tumor-size 50** – dimensions of initial tumor size

**--days** **21** – number of days of simulation

**--diffusion 4** – diffusion method for cancer tumor simulation (other diffusion methods are used in other ABMs)

**--csv-interval 144** – output recording interval (144 timesteps = 1 day)

**--drug-dynamics 11** – molecular-level drug/receptor interactions (11 = carrier endosome equilibrium bystander)

**--stop-on-tumor-volume 2000** – final volume to end simulation (mm^3^)

**--blood-compartment-method 2** – (2 = 2 compartment PK)

**--init-type 1** – grid initialization (1 = *in vivo*, 2 = *in vitro*)

When using the graphics user interface (GUI) version of the model, you can see and select the cell types available on the grid and their colors in the panel under the ‘Agents’ tab and ‘Show Shapes’. You can also define the time of simulation and see the specified parameters in the ‘Simulation’ tab. When the simulation is running, you can see the current time of simulation in the upper right-hand corner of the simulation grid.
